# Vivisection Legislation

**Published:** 1912-10

**Authors:** 


### BUFFALO MEDICAL JOURNAL

**A Monthly Review of Medicine and Surgery**

*EDITOR*

A. L. BENEDICT.

All communications, whether of a literary or business nature, books for review and exchanges, should be addressed to the editor, **228 Summer Street, Cor. Elmwood Avenue, Buffalo, N. Y. Subscribers, advertisers and exchanges please revise address.** Please make personal or telephonic calls before 1 p. m.

THE BUFFALO MEDICAL JOURNAL will publish regularly. full transactions of any professional organization in western New York at cost. Personal notices, brief reports of society proceedings, and the like, are always welcome and will be published without charge.

Subscriptions may commence at any time. $2.00 per year in advance.

VOL. LXVIII. OCTOBER, 1912 No. 3

*Ethics is squareness with the corners projecting.*

### Vivisection Legislation.

Several bills were presented in the New York legislature during the last session, aiming to regulate vivisection, but none was passed. Without pretending to statistic accuracy, the attitude of the antivivisectionists may be gauged by the fact that one or more bills of this nature have been at least prepared for presentation and usually have reached committees, at nearly every session of the legislature, for the last ten years. Many of these bills seem to have been prepared in a conservative and sincere spirit and would appear reasonable to the majority of persons not impressed with the necessity of leaving scientific investigators absolutely unhampered. We cannot help thinking that the atttitude of the profession in antagonizing such legislation, may eventuate in the passage of a drastic bill which will seriously impede scientific investigation and even the practical development of diagnostic and therapeutic measures which depend on no more suffering of animals than their utilization for ordinary economic purposes.

It is in no spirit of hostility to scientific and humanitarian progress, therefore, that we venture to suggest that it might be wise to cooperate with the framer of the next conservative bill, insisting on proper professional representation and control on whatever board of inquiry or of regulation is provided, and suggesting such modifications as may be necessary.

This is a legislative age. Rightly or wrongly—and we may confess to the personal preference for the latter word—the legislative and legal idea, with popular approval, seems to be to codify our method of dealing with every problem that has arisen or may arise. For example there is already under way, a considerable mass of laws, national and international, dealing with aerial navigation and providing for details that, five years ago would have been considered ridiculously impossible and that are still far from practical realization. Vivisection is obviously a practice that is subject to abuse and which has, indeed been abused in the past. It is, therefore, a matter on which the legislative spirit of the age almost inevitably will demand legislation. The present general law regarding cruelty to animals is entirely satisfactory to the medical profession. If we look at the question impartially, we must admit that the law is satisfactory because it is absolutely inefficient, so far as professional vivisectors are concerned. It is conceivable that, under the present law, a perfectly proper and even painless experiment by a professional student or teacher without a medical degree, might be ruled to be a punishable offense. It has been stated by some critics, viewing the present law from the legal side, that it would be possible to punish a physician, engaged in scientific investigation of the highest order, operating with an adequate equipment, and taking every precaution against the infliction of pain, if he were not connected with some chartered institution; and that, on the other hand, the most wanton curelty and neglect, even sadism, on the part of a worker in an institution, would be unpunishable. We are inclined to doubt, however, whether this technical possibility of miscarriage of justice, would ever occur.

However, if susceptible to such construction, even in theory, the present law should be modified in the interests of independent investigators, with or without a medical degree, the sole requirement being a reasonable standard of establishing competence and good faith. On the other hand, it is doubtful whether any future law on vivisection can be so worded as to exempt professional vivisectors from supervision. Such exemption would be contrary to precedent. For example, the liquor law does not exempt professional dealers in liquor; the laws regarding the use of highways do not exempt chauffeurs; in general, laws are made to apply to the persons engaged in the practice which is the subject of regulation.

Shall the profession seize the opportunity to secure a reasonable law by friendly if not entirely complaisant cooperation, or shall it simply obstruct and run the risk of serious interference with scientific and humantarian advance?

